# Effects of hyperbaric oxygen on the osteogenic differentiation of mesenchymal stem cells

**DOI:** 10.1186/1471-2474-15-56

**Published:** 2014-02-25

**Authors:** Song-Shu Lin, Steve WN Ueng, Chi-Chien Niu, Li-Jen Yuan, Chuen-Yung Yang, Wen-Jer Chen, Mel S Lee, Jan-Kan Chen

**Affiliations:** 1 Institute of Biomedical Sciences, Chang Gung University, Taoyuan, Taiwan; 2 Department of Physiology, College of medicine, Chang Gung University, 259 Wen-Hwa 1st Road, Kweishan, 333 Taoyuan, Taiwan; 3 Department of Orthopaedic, Chang Gung Memorial Hospital, No 5, Fu-Hsing Street, 333 Taoyuan, Taiwan

**Keywords:** Hyperbaric oxygen, Mesenchymal stem cells, Wntless, Retromer trafficking protein, Vacuolar ATPases

## Abstract

**Background:**

Hyperbaric oxygenation was shown to increase bone healing in a rabbit model. However, little is known about the regulatory factors and molecular mechanism involved.We hypothesized that the effect of hyperbaric oxygen (HBO) on bone formation is mediated via increases in the osteogenic differentiation of mesenchymal stem cells (MSCs) which are regulated by Wnt signaling.

**Methods:**

The phenotypic characterization of the MSCs was analyzed by flow cytometric analysis. To investigate the effects of HBO on Wnt signaling and osteogenic differentiation of MSCs, mRNA and protein levels of Wnt3a, beta-catenin, GSK-3beta, Runx 2, as well as alkaline phosphatase activity, calcium deposition, and the intensity of von Kossa staining were analyzed after HBO treatment. To investigate the effects of HBO on Wnt processing and secretion, the expression of Wntless and vacuolar ATPases were quantified after HBO treatment.

**Results:**

Cells expressed MSC markers such as CD105, CD146, and STRO-1. The mRNA and protein levels of Wnt3a, β-catenin, and Runx 2 were up-regulated, while GSK-3β was down-regulated after HBO treatment. Western blot analysis showed an increased β-catenin translocation with a subsequent stimulation of the expression of target genes after HBO treatment. The above observation was confirmed by small interfering (si)RNA treatment. HBO significantly increased alkaline phosphatase activity, calcium deposition, and the intensity of von Kossa staining of osteogenically differentiated MSCs. We further showed that HBO treatment increased the expression of Wntless, a retromer trafficking protein, and vacuolar ATPases to stimulate Wnt processing and secretion, and the effect was confirmed by siRNA treatment.

**Conclusions:**

HBO treatment increased osteogenic differentiation of MSCs via regulating Wnt processing, secretion, and signaling.

## Background

Hyperbaric oxygen (HBO) therapy is a safe noninvasive modality that increases the oxygen tension of tissues and microvasculature [1]. HBO increases the expression of Wnt-3 protein in neural stem cells [2]. A previous study suggested that Wnt signaling could stimulate bone healing [3]. We previously reported the beneficial effects of HBO on bone lengthening in a rabbit model [4]. However, little is known about the effects of HBO on the osteogenesis and Wnt signaling in mesenchymal stem cells (MSCs).

Wnt proteins are secreted lipid-modified signaling molecules that influence animal development [5]. The target cells for the Wnt proteins expressed by MSCs may be either MSCs themselves or other cell types in the bone marrow [6]. On target cells, secreted Wnt protein interacts with the receptors Frizzled and LRP5/6 to activate the β-catenin pathway [7]. Activation of the Frizzled receptor complex results in the inhibition of a phosphorylation cascade that stabilizes intracellular β-catenin levels. β-Catenin is subsequently translocated into the nucleus to regulate the Wnt target genes. In the absence of Wnt protein, β-catenin is phosphorylated by glycogen synthase kinase–3β (GSK-3β) and subsequently degraded by proteasomes. Among Wnt family members, Wnt3a is involved in the proliferation and differentiation of MSCs [8]. Runx2, a member of the *runt* homology domain transcription factor family, is essential for osteoblast differentiation [9]. Canonical Wnt signaling promotes osteogenesis by directly stimulating *Runx2* gene expression and this regulation can be antagonized by secreted frizzled-related protein-1 (SFRP1) [10].

In Wnt producing cells, endosomal transport and acidification are essential functions in Wnt processing and secretion [11,12]. Wnt is synthesized and lipid modified in the endoplasmic reticulum (ER). It is then transported to the Golgi complex, where it binds to Wntless (Wls). Wls supports the transport of Wnt from the trans-Golgi network (TGN) to the cell surface in vesicles, from which Wnt is then released. After Wnt is released, Wls is internalized through AP-2/clathrin-mediated endocytosis. The retromer complex interacts with Wls and retrieves Wls from endosomes back to TGN, thereby maintaining the normal levels of Wls protein [11]. The core of the retromer complex consists of the VPS35, VPS26, and VPS29 subunits [13]. In the absence of retromer activity, internalized Wls is likely to be sorted into lysosomes and then degraded [14,15]. Wls becomes unstable in the absence of retromer activity, and mutant retromer inhibited Wnt signaling [14-16]. The overexpression of retromer can significantly enhance levels of Wls in mammalian cells even in the absence of the Wnt ligand [16]. The mammalian ortholog of Wls is GPR177, a putative orphan G protein–coupled receptor (GPCR) [17]. GPR177 has been shown to regulate Wnt protein secretion in cells [15,18].

Vacuolar acidification is required for Wnt signaling [12,19]. Vacuolar ATPases (V-ATPases) are large multisubunit complexes that are organized into 2 domains that operate by a rotary mechanism. The V1 domain is located on the cytoplasmic side of the membrane, and carries out ATP hydrolysis. The V0 domain is a membrane-embedded complex that is responsible for translocating protons from the cytoplasm to the extracellular space [20]. V-ATPase-driven proton pumping and organellar acidification are essential for vesicular trafficking along both the exocytotic and endocytotic pathways of eukaryotic cells. The inhibition of V-ATPase results in accumulation of the Wnt3a-Wls complex, inhibits the release of Wnt3a, and inhibits Wnt/β-catenin signaling both in cultured human cells and in vivo [19].

In the present study, we found that the beneficial effect of HBO on the osteogenesis of MSCs is regulated via Wnt signaling pathway. Science endosomal transport and acidification are essential functions in Wnt secretion, we further showed that HBO treatment increased the expression of GPR177, VPS35, and V-ATPases to stimulate Wnt processing and secretion.

## Methods

The experimental protocol was performed in accordance with the Declaration of Helsinki and approved by the human subjects Institutional Review Board of the Chang Gung Memorial Hospital. Written informed consent was obtained from all patients. Demographic and clinical data such as age, gender and surgery reason were collected.

### Patients and surgical procedures

MSCs were harvested from 12 patients (5 females and 7 males) who underwent iliac bone grafting for spine fusion. The mean age was 58.3 years-old, where the age range from 39 to 77 years old. The cells from each patient were separately evaluated. The cells from 3 or 4 patient were subjected to each treatment. During bone graft harvesting, 10 mL of bone marrow was aspirated and collected in a sterile heparin-rinsed syringe.

### Isolation and cultivation of MSCs

Each marrow sample was washed with PBS. Up to 2 × 10^8^ nucleated cells in 5 mL of PBS were loaded onto 25 mL of Percoll cushion (Pharmacia Biotech). A density gradient was used as the isolation procedure to eliminate unwanted cell types. A small percentage of cells were isolated from the density interface at 1.073 g/mL. The cells were re-suspended and plated at 2 × 10^5^ cells per T-75 flasks. The cells were maintained in Dulbecco's Modified Eagle's Medium-Low Glucose (DMEM-LG; Gibco, Grand Island, NY) that containing 20% fetal bovine serum (FBS) and antibiotics at 37°C in a humidified atmosphere of 5% CO_2_ and 95% air. After 7 d of primary culturing, the non-adherent cells were removed by changing the medium. The MSCs grew as symmetric colonies and were subcultured at 10 to 14 d by treatment with 0.05% trypsin (Gibco) and seeded into fresh flasks.

### Flow cytometric analysis of surface antigen expression

When confluent, the MSCs were passaged 1 in 3, and a sample was analyzed for MSCs marker expression by flow cytometry. The cells were washed in phosphatebuffered saline (PBS), and then removed from the flask by 0.05% trypsin (Gibco). 1 × 10^5^ cells were incubated with each mouse monoclonal primary antibody at 4°C for 30 minutes. Mouse FITC–conjugated anti-CD105 antibody (1:100 dilution), mouse PE–conjugated anti-CD146 antibody (1:100 dilution), and mouse FITC–conjugated anti-CD34 antibody (1:100 dilution) were purchased from Beckton Dickinson (Oxford, UK). Mouse PE–conjugated anti-STRO-1 antibody (1:50 dilution) was purchased from Santa Cruz (CA, USA). After wash, the cells were resuspended in 500 μl wash buffer and analyzed on a BD flow cytometer (Oxford, UK).

### Cell exposure to intermittent HBO

The cells were cultured in 100 mm culture dishes (2 × 10^5^ per dish) in complete medium (DMEM-LG containing 20% FBS and antibiotics) or in osteogenic induction medium (DMEM-LG containing 20% FBS, antibiotics, 100 μM ascorbate-2 phosphate, 100 nM dexamethasone, and 10 mM β-glycerophosphate). The cells were either maintained in 5% CO_2_/95% air throughout the experiment or were HBO treated by exposure to 100% O_2_ for 25 min and then to 5% CO2/95% air for 5 min at 2.5 ATA (atmospheres absolute) in a hyperbaric chamber (Huxley Corporation, Taipei, Taiwan) for 90 min every 36 h.

### RNA preparation and real-time quantitative polymerase chain reaction analysis

After culturing for 1, 4, and 7 d with or without HBO treatment, total RNA was extracted using a Qiagen RT kit (Qiagen, USA) according to the manufacturer’s instructions. The RNA concentration was evaluated by A260/A280 measurement. To detect Wnt3a, GSK-3β, β-catenin, Runx2, and GAPDH RNA transcripts, cDNA was analyzed on an ABI PRISM 7900 sequence detection system using TaqMan PCR Master Mix (Applied Biosystems, Foster City, CA). The cycle threshold (Ct) values were obtained, and the data were normalized to GAPDH expression by using the ΔΔCt method to calculate the relative mRNA level of each target gene.

### Small interfering RNA transfection

On day 1, 2 × 10^5^ MSCs were plated onto a 6-well tissue culture plate in 2.5 mL of OPTI-MEM (Invitrogen, Carlsbad, CA) medium without antibiotics and serum. The cells were then transfected with human β-catenin small interfering (si)RNA or scrambled siRNA (Stealth RNAi, Invitrogen) using Lipofectamine RNAiMAX (Invitrogen) according to the manufacturer’s instructions. After 8 h of transfection, the culture medium was changed to osteogenic medium with 10% FBS and the cells were exposed to HBO treatment. On days 4 and 7, the cells were re-transfected once and exposed to HBO. After an additional 24 h of culturing, the cells were harvested for analysis. The silencing effect on β-catenin and downregulation of Runx 2 was detected by real-time PCR after the treatments.

### Western blot analysis

After culturing for 7 d with or without HBO treatment, the cells were washed with PBS and extracted using M-PER mammalian protein extraction reagent (Thermo, USA). The protein content was quantitated using a protein assay kit (Pierce Biotechnology, IL), separated by 7.5% SDS-PAGE for Wnt3a, GSK-3β, β-catenin, Runx2, β-actin, and α-tubulin, and transferred onto membranes using a transfer unit (Bio-Rad, USA). After blocking with 10% non-fat milk, the membranes were incubated overnight at 4°C with 1000-fold diluted rabbit antibodies against Wnt3a, GSK-3β (Cell Signaling, MA, USA) or mouse antibodies against β-catenin (Millipore), β-actin (Millipore), and Runx2 (Millipore). After washing, the membranes were further incubated for 2 h with 10000-fold goat anti-mouse IgG (Calbiochem, USA) or goat anti-rabbit IgG (Millipore) conjugated to horseradish peroxidase. The membranes were then washed and rinsed with ECL detection reagents (Amersham Pharmacia Biotech, UK). The band images were photographed using ECL Hyperfilm (Amersham). The intensity of each stained was quantified using an image-analysis system (Image-Pro plus 5.0, Media Cybernetics, USA).

### Preparation of cytosolic and nuclear fractions for β-catenin detection

After culturing for 7 d with or without HBO treatment, the cells were rinsed with ice cold PBS, treated with 0.05% trypsin, and then collected by centrifugation at 800 g. NE-PER nuclear and cytoplasmic extraction reagents (Thermo science, USA) were used to isolate cytoplasmic and nuclear extracts from the cells. The protein content was quantitated using a protein assay kit (Pierce), and separated by 7.5% SDS-PAGE to detect β-catenin (Millipore) and TATA binding protein (TBP; Abcam, Cambridge, UK). The silencing effect on β-catenin and downregulation of Runx 2 was detected by western blotting after the treatments.

### Quantitative measurement of alkaline phosphatase activity

After culturing for 7, 14, and 21 d with or without HBO treatment, the cultured cells were washed with ice cold PBS. A 5-mL of alkaline phosphatase (ALP) substrate buffer (50 mM glycine, 1 mM MgCl_2_, pH 10.5), containing soluble ALP substrate (2.5 mM *p*-nitrophenyl phosphate), was added at room temperature. Twenty minutes after adding the substrate, 1 mL of the buffer was removed from the culture and mixed with 1 mL of 1 N NaOH to halt each reaction. The absorbance of each mixture was determined on an ELISA plate-reader (MRX; Dynatech Labs) at 405 nm. Enzyme activity was expressed as n mole p-nitrophenol/min.

### Calcium level quantification

After culturing for 7, 14, and 21 d with or without HBO treatment, the cultured cells were rinsed with ice cold PBS and placed into 5 mL of 0.5 N HCl. Calcium was extracted from the cells by gently shaking the cultures for 24 h. Cellular debris was centrifuged and the calcium in the supernatant was measured using a Quantichrom calcium assay kit (DICA-500, Bioassay systems, USA).

### von Kossa staining

After culturing for 21 d with or without HBO treatment, culture dishes were rinsed twice with 5 mL of Tyrode’s balanced salt solution, and fixed in 10% buffered formalin for 1 h. A 10-mL aliquot of freshly prepared 2% (w/v) silver nitrate in water was added, and the dishes were kept in dark for 30 min. The plates were then washed thoroughly with distilled water and exposed to bright light for 30 min. The presence of mineral deposits was indicated by the development of a black precipitate on the mineralized matrix. The matrix intensity was quantified by image-analysis system (Image-Pro plus 5.0).

### Dedicated Wnt secretion factors assay

After culturing for 1, 4, and 7 d with or without HBO treatment, the culture medium was collected and the cells were washed with ice cold PBS and cellular protein was extracted using M-PER protein extraction reagent (Thermo, USA). Each protein extraction was separated by 7.5% SDS-PAGE to detect the GPR177 (Millipore), VPS35 (Abcam), ATP6V0 (Abcam), Wnt3a (Millipore), and β-actin (Millipore). The secreted Wnt3a in the collected medium was quantified by ELISA (USCN Life Science, Wuhan, PR China).

### RNAi treatment for GPR177, VPS35, and V-ATPases

MSCs were transfected with siRNA for GPR177, VPS35, and ATP6V0 (Santa Cruz), respectively on days 1, 4, and 7 by using the same protocol above described. Silencing was detected by western blotting after the treatments. The secreted Wnt3a in the collected medium was quantified by ELISA (USCN).

### Statistical analysis

Data are given as mean ± standard devision (SD) of the results from three or four different samples in each item of the experiment. The cells from each sample were separately evaluated. Differences between two groups were measured by the Student’s *t*-test. A *p* value less than 0.05 was defined statistically significant difference.

## Results

### Flow cytometry analysis

**Figure 1:**
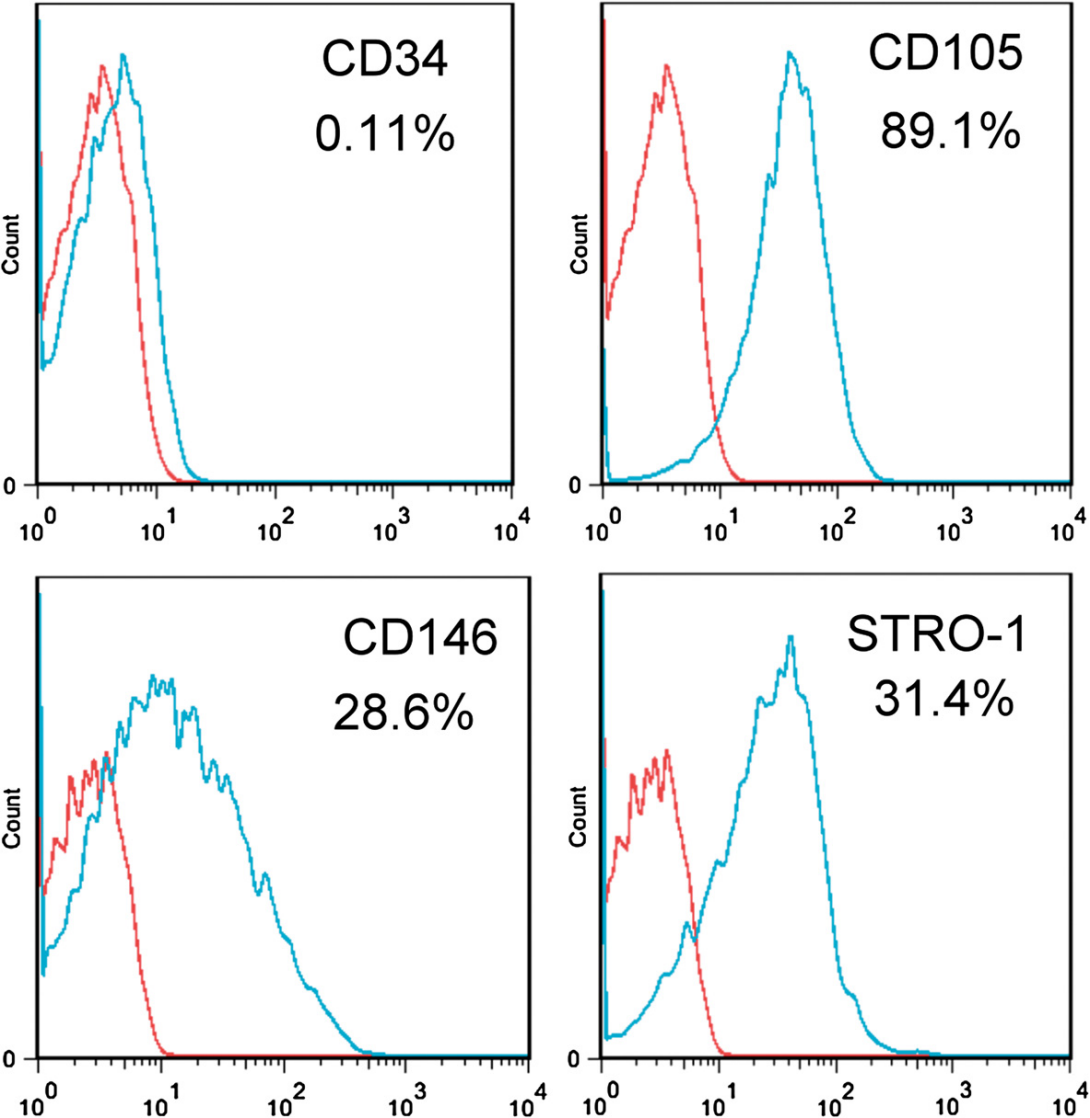
**Flow cytometry analysis of passage 1 cells from 1 patient.** The percentage of cells expressing the MSC markers CD146, CD105, and Stro-1 and hematopoietic cell marker CD34 were shown. The blue line areas represent the distribution of cells stained by the respective antibodies; the red line areas are control cells without staining. Percentage numbers indicate the percentages of cells positively stained by the respective antibodies in the flow cytometry analysis.

Primary adherent human MSCs from 4 donors were cultured in control medium, and cells were analyzed for expression of MSC markers using flow cytometry at passage 1. The percentage of cells expressing the MSC markers CD146, CD105, and Stro-1 and hematopoietic cell marker CD34 were shown in Figure 1. The mean percentages of CD146^+^, CD105^+^, Stro-1^+^, and CD34^+^ cells in the cell preparations from 4 patients (n = 4) were calculated to be 28.2% ± 1.66%, 90.0% ± 1.94%, 32.3% ± 0.89%, and 0.10% ± 0.03%, respectively.

### Effect of HBO on mRNA expression by MSCs

**Figure 2:**
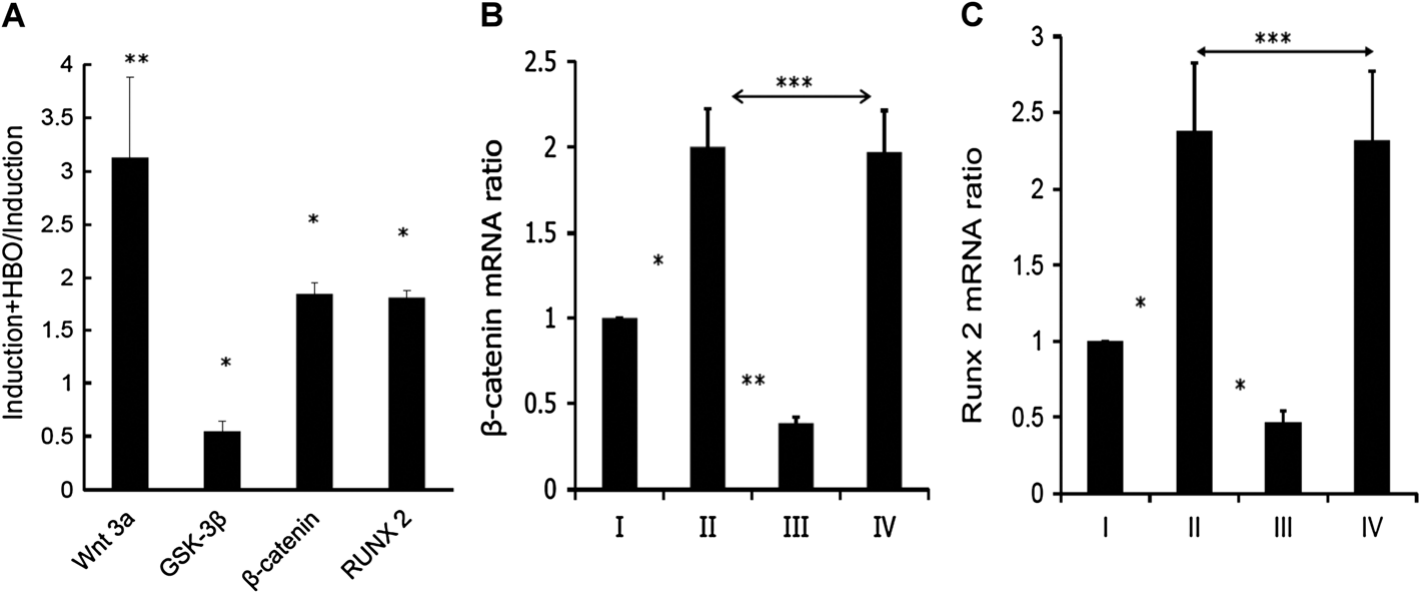
**Effect of HBO on mRNA expression by MSCs.** The mRNA ratios of Wnt3a (**A**, II / I, **p < 0.01, n = 3, Student’s *t*-test), β-catenin (**A**, II / I, *p < 0.05, n = 3, Student’s *t*-test), and Runx2 (**A**, II / I, *p < 0.05, n = 3, Student’s *t*-test) were up-regulated, while GSK-3β (**A**, II / I, *p < 0.05, n = 3, Student’s *t*-test) was down-regulated after HBO treatment. The mRNA levels of β-catenin (**B**, II / I, *p < 0.05, n = 4, Student’s *t*-test) and Runx2 (**C**, II / I, *p < 0.05, n = 4, Student’s *t*-test) were up-regulated after HBO treatment. The silencing effect on β-catenin (**B**, II vs. III, **p < 0.01, n = 4, Student’s *t*-test) and downregulating effect for Runx2 (**C**, II vs. III, *p < 0.05, n = 4, Student’s *t*-test) by β-catenin siRNA were detected after the treatments. No significant difference was shown between II and IV (***p > 0.05, n = 4, Student’s *t*-test, **B, C**). (I, induction; II, induction + HBO; III, Induction + HBO + siRNA; IV, Induction + HBO + scrambled siRNA).

Real-time Q-PCR data showed that the mRNA ratios of Wnt3a (II / I, 3.16 ± 0.72-fold on D7, **p < 0.01), β-catenin (II / I, 1.85 ± 0.1-fold on D7, *p < 0.05), and Runx2 (II / I, 1.81 ± 0.07-fold on D7, *p < 0.05) were up-regulated, while GSK-3β (II / I, 0.55 ± 0.09-fold on D7, *p < 0.05) was down-regulated after HBO treatment (Figure 2A). The mRNA levels of β-catenin (II / I; 2.0 ± 0.22-fold, *p < 0.05, Figure 2B), and Runx2 (II / I; 2.37 ± 0.44-fold, *p < 0.05, Figure 2C) were up-regulated after HBO treatment. The silencing effect on β-catenin (II / I vs. III / I, 2.0 ± 0.22-fold vs. 0.39 ± 0.04-fold, **p < 0.01, Figure 2B) and downregulating effect for Runx2 (II / I vs. III / I, 2.37 ± 0.45-fold vs. 0.46 ± 0.07-fold, *p < 0.05, Figure 2C) by β-catenin siRNA were detected after the treatments. No significant difference was shown by scrambled siRNA treatment (II / I vs. IV / I, 2.0 ± 0.22-fold vs. 1.97 ± 0.24-fold, ***p > 0.05, Figure 2B; II / I vs. IV / I, 2.37 ± 0.45-fold vs. 2.31 ± 0.46-fold, ***p > 0.05, Figure 2C) (I, induction; II, induction + HBO; III, induction + HBO + siRNA; IV, induction + HBO + scrambled siRNA).

### Effect of HBO on protein expression by MSCs

**Figure 3:**
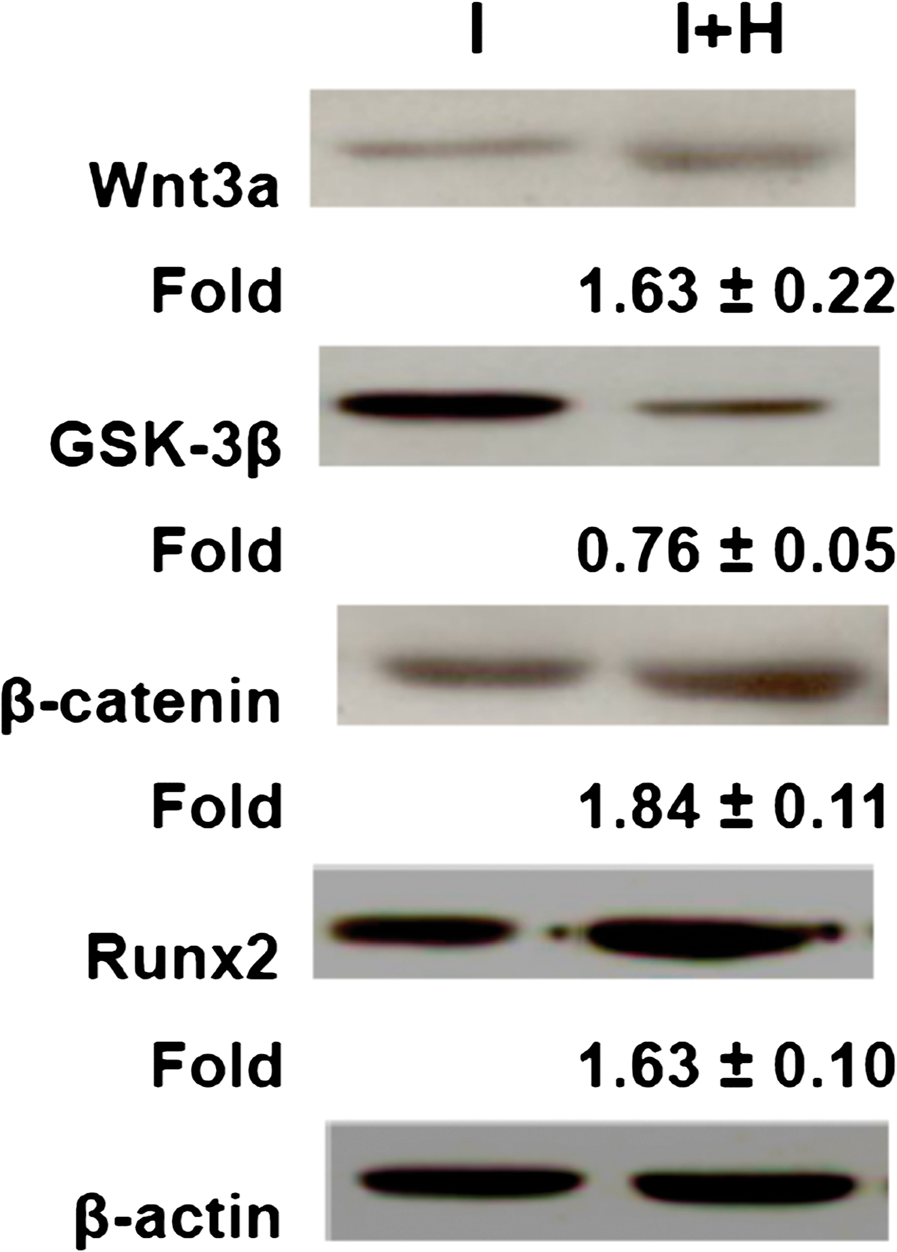
**Effect of HBO on protein expression by MSCs.** The western blot data showed that the protein levels of Wnt3a (p < 0.05, n = 4, Student’s *t*-test), β-catenin (p < 0.05, n = 4, Student’s *t*-test) and Runx2 (p < 0.05, n = 4, Student’s *t*-test) were upregulated but GSK-3β (p < 0.05, n = 4, Student’s *t*-test) was downregulated after HBO treatment.

**Figure 4:**
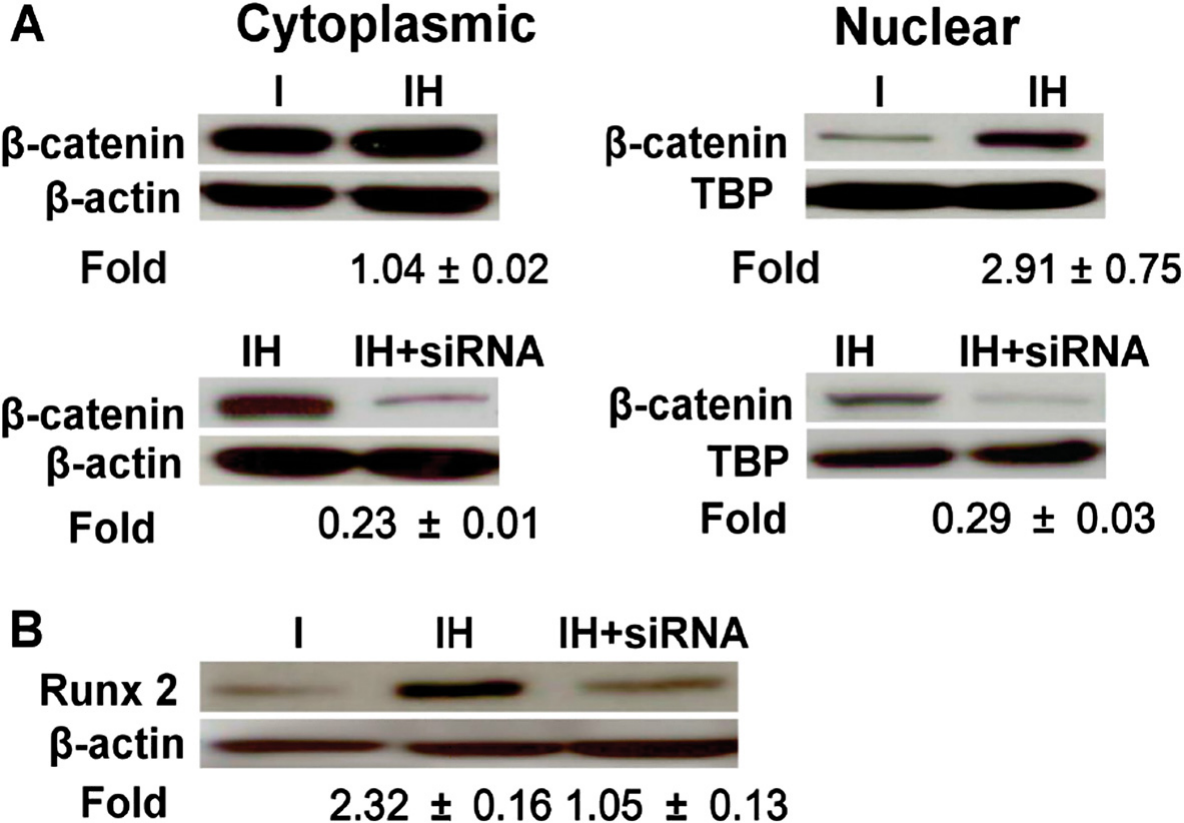
**Effect of HBO on β-catenin translocation in MSCs.** The protein levels of β-catenin in the nuclear fractions were up-regulated after HBO treatment (2.91 ± 0.75-fold, p < 0.05, n = 3, Student’s *t*-test, **A)**. No significant difference in the cytoplasmic fractions was shown after HBO treatment (1.04 ± 0.22-fold, p > 0.05, n = 3, Student’s *t*-test, **A)**. The increased protein levels of β-catenin **(A)** and Runx2 **(B)** by HBO treatment were all down-regulated by β-catenin siRNA treatment.

The Western blot data showed that the protein levels of Wnt3a (1.63 ± 0.22-fold, p < 0.05), β-catenin (1.84 ± 0.11-fold, p < 0.05) and Runx2 (1.63 ± 0.10-fold, p < 0.05) were upregulated but GSK-3β (0.76 ± 0.05-fold, p < 0.05) was downregulated after HBO treatment (Figure 3). HBO increased the osteogenic differentiation of the MSCs and the Wnt pathway signaling. We found that the β-catenin protein levels in the nuclear fractions were up-regulated after HBO treatment (2.91 ± 0.75-fold, p < 0.05, Figure 4A). In addition, the Runx2 protein levels were up-regulated after HBO treatment (2.32 ± 0.16-fold, p < 0.05, Figure 4B). HBO treatment increased the translocation of β-catenin from the cytosol into the nucleus. To explore whether the effect of HBO on Runx2 expression was via translocation of β-catenin, the cells were transfected with siRNA against β-catenin. We found that the increased β-catenin (Figure 4A) and Runx2 (Figure 4B) protein levels by HBO treatment were all down-regulated by β-catenin siRNA treatment.

### Long-term effects of HBO on MSCs

**Figure 5:**
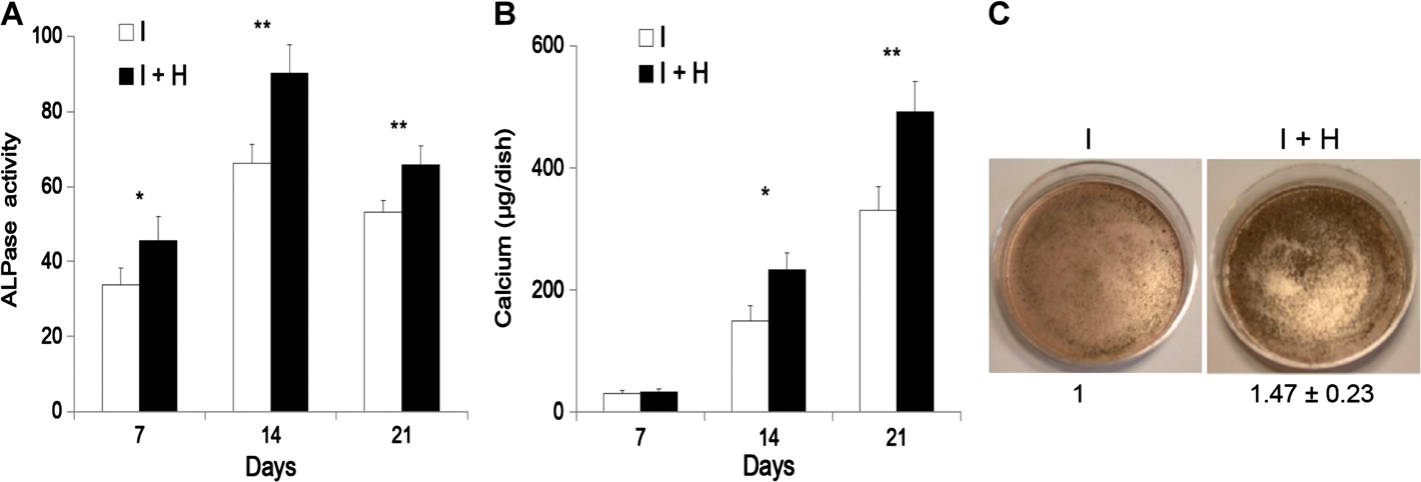
**Long-term effects of HBO on MSCs. (A)** HBO significantly increased alkaline phosphatase activity after 7 d (*p < 0.05, n = 4, Student’s *t*-test), 14 d (**p < 0.01, n = 4, Student’s *t*-test), and 21 d (**p < 0.01, n = 4, Student’s *t*-test) of culturing. **(B)** HBO significantly increased calcium levels after 14 d (*p < 0.05, n = 4, Student’s *t*-test) and 21 d (**p < 0.01, n = 4, Student’s *t*-test) of culturing. **(C)** Greater positive von Kossa staining through the matrix at the surface layer of the HBO group was observed compared to the control group (1.473 ± 0.23-fold, p < 0.05, n = 3, Student’s *t*-test).

HBO significantly increased ALP activity after 7 d (33.7 ± 4.5 vs. 45.6 ± 6.5, *p < 0.05), 14 d (66.3 ± 5.1 vs. 90.2 ± 7.6, **p < 0.01), and 21 d (53.2 ± 3.2 vs. 65.9 ± 5.1, **p < 0.01) of culturing (Figure 5A). The ALP activity increased was coincided with the increase of calcium levels after 14 d (149.1 ± 25.4 vs. 234.0 ± 26.7, *p < 0.05) and 21 d (331.0 ± 38.7 vs. 492.8 ± 48.4, **p < 0.01) of culturing (Figure 5B) in the osteogenic induction medium. The deposition of a calcified matrix on the surface of the culture dish was evident by von Kossa staining. The matrix intensity for the induction + HBO group and induction group were 557505.3 ± 55457.4 and 382909.7 ± 55873.8 which were quantified by image-analysis system. Greater positive staining through the matrix at the surface layer of the induction + HBO group was observed compared to the induction group (1.47 ± 0.23-fold, p < 0.05, Figure 5C).

### Effect of HBO on GPR177 and Wnt3a protein levels

**Figure 6:**
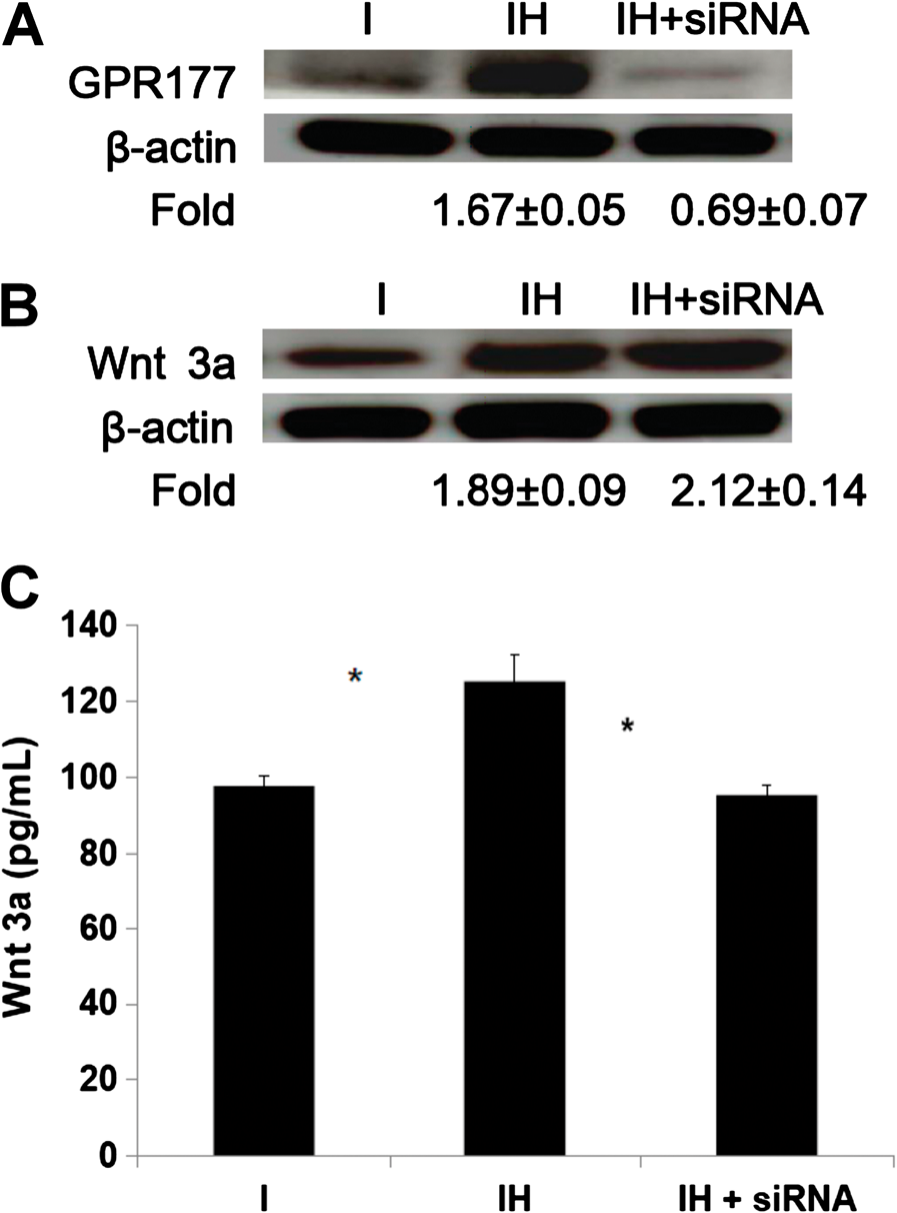
**Effect of HBO on the expression of GPR177 and Wnt3a.** Protein levels of GPR177 were up-regulated after HBO treatment in the cell lysates (1.67 ± 0.05-fold, p < 0.01, **A)** and the effect of HBO was reduced through GPR177 siRNA treatment (1.67 ± 0.05-fold vs. 0.69 ± 0.07-fold, p < 0.01, **A)**. The amounts of Wnt3a in the cell lysates (1.89 ± 0.09-fold, p < 0.05, **B)** and conditioned culture medium (I vs. IH, *p < 0.05, **C)** were both up-regulated after HBO treatment. However, the effect of HBO on secreted Wnt3a was reduced (IH vs. IH + siRNA, *p < 0.05, **C)**, but the intracellular Wnt3a levels were not affected (IH/I vs. IH + siRNA/I, p > 0.05, **B)** by GPR177 siRNA treatment. Each bar represents the value of the mean ± SD and analyzed by Student t-test (I, induction; IH, induction + HBO).

Protein levels of GPR177 **(**Wls) were up-regulated after HBO treatment (IH/I, 1.67 ± 0.05-fold, p < 0.01, n = 3, Student’s *t*-test,) and the effect of HBO was reduced after GPR177 siRNA treatment (IH/I vs. IH + siRNA/I; 1.67 ± 0.05-fold vs. 0.69 ± 0.07-fold, p < 0.01, n = 3, Student’s *t*-test; Figure 6A). The Wnt3a levels in the cell lysates (IH/I, 1.89 ± 0.09-fold, p < 0.05, n = 3, Student’s *t*-test; Figure 6B) and conditioned culture medium (I vs. IH; 97.7 ± 2.8 pg/mL vs. 125.3 ± 7.2 pg/mL, *p < 0.05, n = 3, Student’s *t*-test; Figure 6C) were both up-regulated after HBO treatment. Interestingly, the effect of HBO on secreted (Figure 6C), but not intracellular Wnt3a (Figure 6B), was reduced by GPR177 siRNA treatment (IH/I vs. IH + siRNA/I; 1.89 ± 0.09-fold vs. 2.12 ± 0.14-fold, p > 0.05, n = 3, Student’s *t*-test; Figure 6B), ( IH vs. IH + siRNA; 125.3 ± 7.2 pg/mL vs. 95.4 ± 2.5 pg/mL, *p < 0.05, n = 3, Student’s *t*-test; Figure 6C) (I, induction; IH, induction + HBO).

### Effect of HBO on the expression of VPS35 and Wnt3a

**Figure 7:**
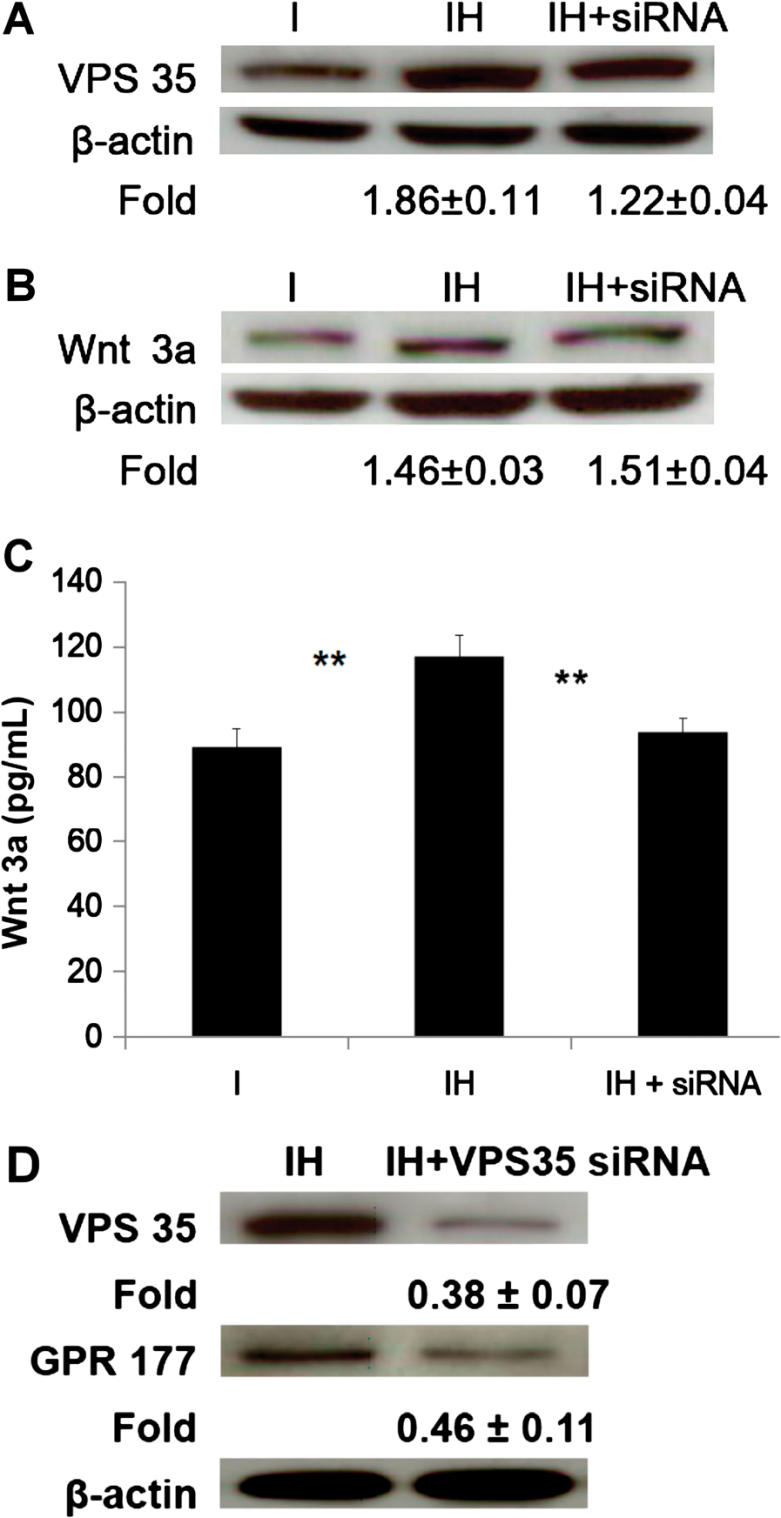
**Effect of HBO on the expression of VPS35 and Wnt3a.** Protein levels of VPS35 were up-regulated after HBO treatment in the cell lysates (1.86 ± 0.11-fold, p < 0.01, **A)** and the effect of HBO was reduced by VPS35 siRNA treatment (1.86 ± 0.11-fold vs. 1.22 ± 0.04-fold, p < 0.05, **A)**. The amounts of Wnt3a in the cell lysates (1.46 ± 0.03-fold, p < 0.05, **B)** and conditioned culture medium (I vs. IH, **p < 0.01, **C)** were both up-regulated after HBO treatment. The effect of HBO on secreted Wnt3a was reduced (IH vs. IH + siRNA,**p < 0.01, **C)** but the intracellular Wnt3a levels were not affected (IH/I vs. IH + siRNA/I, p > 0.05, **B)** by VPS35 siRNA treatment. GPR177 level was reduced (0.46 ± 0.11-fold, p < 0.01, **D)** when VPS35 was suppressed (0.38 ± 0.07-fold, p < 0.01, **D)** by VPS35 siRNA treatment. Each bar represents the value of the mean ± SD and analyzed by Student t-test (I, induction; IH, induction + HBO).

Protein levels of VPS35 (retromer subunit) were up-regulated after HBO treatment in the cell lysates (IH/I, 1.86 ± 0.11-fold, p < 0.01, n = 3, Student’s *t*-test) and the effect of HBO was reduced through VPS35 siRNA treatment (IH/I vs. IH + siRNA/I; 1.86 ± 0.11-fold vs. 1.22 ± 0.04-fold, p < 0.05, n = 3, Student’s *t*-test; Figure 7A). The amounts of Wnt3a in the cell lysates (IH/I; 1.46 ± 0.03-fold, p < 0.05, n = 3, Student’s *t*-test; Figure 7B) and conditioned culture medium (I vs. IH; 89.2 ± 5.6 pg/mL vs. 117.1 ± 6.4 pg/mL, **p < 0.01, n = 3, Student’s *t*-test; Figure 7C) were both up-regulated after HBO treatment. However, the effect of HBO on secreted Wnt3a was reduced (IH vs. IH + siRNA; 117.1 ± 6.4 pg/mL vs. 93.6 ± 4.2 pg/mL, **p < 0.01, n = 3, Student’s *t*-test; Figure 7C), but the intracellular Wnt3a levels were not affected (IH/I vs. IH + siRNA/I; 1.46 ± 0.03-fold vs. 1.51 ± 0.04-fold, p > 0.05, n = 3, Student’s *t*-test; Figure 7B) by VPS35 siRNA treatment. The possible effects of VPS35 on GPR177 stability after HBO treatment was examined, we found that GPR177 level was reduced (0.46 ± 0.11-fold, p < 0.01, n = 3, Student’s *t*-test; Figure 7D) when VPS35 was suppressed (0.38 ± 0.07-fold, p < 0.01, n = 3, Student’s *t*-test; Figure 7D) by VPS35 siRNA treatment.

### Effect of HBO on the expression of ATP6V0 and Wnt3a

**Figure 8:**
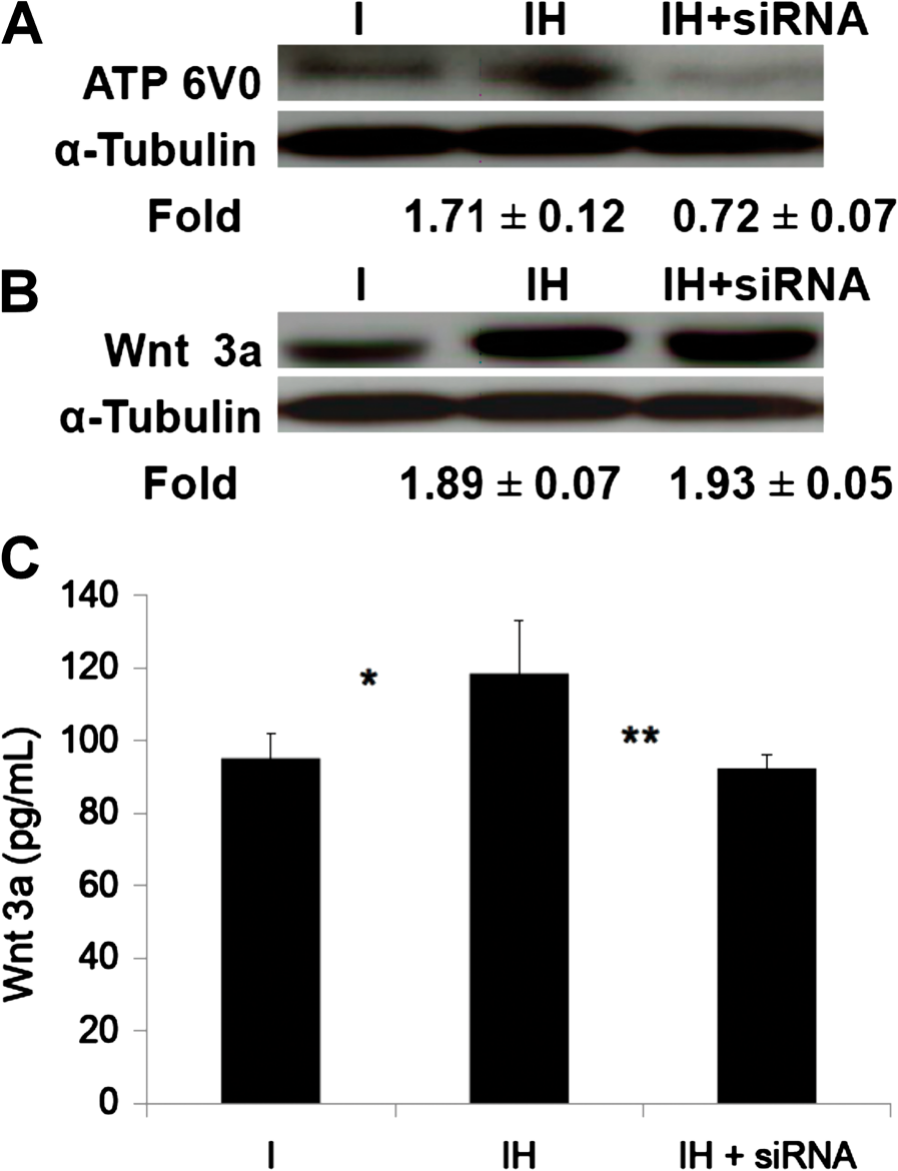
**Effect of HBO on the expression of ATP6V0 and Wnt3a.** Protein levels of ATP6V0 were up-regulated after HBO treatment in the cell lysates (1.71 ± 0.12-fold, p < 0.01, **A)** and the effect of HBO was reduced by ATP6V0 siRNA treatment (1.71 ± 0.12-fold vs. 0.72 ± 0.07-fold, p < 0.01, **A)**. The amounts of Wnt3a in the cell lysates (1.89 ± 0.07-fold, p < 0.01, **B)** and conditioned culture medium (I vs. IH, *p < 0.05, **C)** were both up-regulated after HBO treatment. The effect of HBO on secreted Wnt3a was reduced (IH vs. IH + siRNA, **p < 0.01, **C)** but the intracellular Wnt3a levels were not affected (IH/I vs. IH + siRNA/I, p > 0.05, n = 3; **B)** by ATP6V0 siRNA treatment. Each bar represents the value of the mean ± SD and analyzed by Student t-test (I, induction; IH, induction + HBO).

Protein levels of ATP6V0 were up-regulated after HBO treatment in the cell lysates (IH/I, 1.71 ± 0.12-fold, p < 0.01, n = 3, Student’s *t*-test) and the effect of HBO was reduced by ATP6V0 siRNA treatment (IH/I vs. IH + siRNA/I; 1.71 ± 0.12-fold vs. 0.72 ± 0.07-fold, p < 0.01, n = 3, Student’s *t*-test; Figure 8A). The Wnt3a levels in the cell lysates (IH/I; 1.89 ± 0.07-fold, p < 0.01, n = 3, Student’s *t*-test; Figure 8B) and conditioned culture medium (I vs. IH; 95.1 ± 6.6 pg/mL vs. 118.4 ± 14.7 pg/mL, *p < 0.05, n = 3, Student’s *t*-test; Figure 8C) were both up-regulated after HBO treatment. However, the effect of HBO on Wnt3a levels was reduced in the conditioned medium (IH vs. IH + siRNA; 118.4 ± 14.7 pg/mL vs. 92.3 ± 3.7 pg/mL, **p < 0.01, n = 3, Student’s *t*-test; Figure 8C) but not in the cell lysates (IH/I vs. IH + siRNA/I; 1.89 ± 0.07-fold vs.1.93 ± 0.05-fold, p > 0.05, n = 3, Student’s *t*-test; Figure 8B) after ATP6V0 siRNA treatment.

## Discussion

Low oxygen tension was shown to promote the undifferentiated state and attenuate differentiation capacity of MSCs [21]. MSCs cultured under hypoxia exhibited decreased differentiation into osteogenic cells [22]. MSC osteogenesis was associated with a higher level of oxygen consumption compared with chondrogenesis, and therefore reduced oxygen consumption during the differentiation process may inhibit osteogenesis [16]. Our present findings supported those of previous studies, suggesting that MSCs cultured under HBO undergo increased differentiation into osteogenic cells by up-regulating Runx2 expression (Figure 1A). Because oxygen availability regulates stem cells via Wnt/β-catenin signaling [23], we intended to examine the molecular mechanisms involved after HBO treatment by assessing the Wnt/β-catenin pathway. Our data showed that the levels of Wnt3a, β-catenin, and Runx2 were upregulated, while GSK-3β was downregulated after HBO treatment. HBO increased β-catenin mRNA production to stimulate Runx2 mRNA expression and this was confirmed by β-catenin siRNA treatment (Figure 2B, 2C). In addition, we found that the accumulated β-catenin was subsequently translocated into the nucleus (Figure 4A) where it upregulated Runx2 protein expression and the effect was also confirmed by β-catenin siRNA treatment (Figure 4B).

Osteoblasts originate from MSC via a stepwise maturation process. During the early stage of osteogenesis, the cell cannot deposit calcium to form mineralized bone [9]. In order to deposit calcium, the cells must enter the late stage of osteogenesis [24]. Because we cannot find the short-term effects of HBO (7 d) on calcium production, we therefore, investigated the long-term effects of HBO (14 and 21 d) on the osteogenesis of MSCs and found that HBO significantly increased the expression of osteogenic markers (Figure 5A, B). Enhanced positive matrix von Kossa staining at the surface layer of the HBO group was seen compared to the control group (Figure 5C).

Three independent groups have identified the multi-pass transmembrane protein Wls as a dedicate component required for Wnt secretion [25-27]. In the absence of Wls, some Wnt proteins may sequestered in the secretory pathway in Wnt-producing cells. The protein failed to reach the plasma membrane and surrounding cells, thereby resulting in Wnt loss-of-function phenotypes [14,25]. In the present study, HBO treatment was found increase Wnt3a production, however, Wnt3a was retained in the producing cells (Figure 6B) and was unable to move into culture medium (Figure 6C) during Wls (GPR177) siRNA treatment. Clearly, the secretion of Wnt3a was impaired upon knockdown of GPR177. GPR177 was shown to be a transcriptional target of Wnt/β-catenin signaling in embryonic axis formation [17], thus, Wnt-dependent activation of GPR177 in the MSCs deserved further investigation.

Parallel to the discovery of Wls, several researchers revealed that retromer is also required for Wnt signaling. Their observation suggested that in retromer mutant clones, Wnt accumulates in Wnt-producing cells and that the Wnt concentration gradient created by secretion is strongly diminished [14,15,18]. However, one group suggested that retromer (VPS35) siRNA in mammalian cells impairs Wnt signaling but not Wnt secretion [28]. Although Wnt3a production was increased by HBO treatment, our data were consistent with previous studies which suggested that Wnt3a is retained in the producing cells (Figure 7B) and is unable to secret into the culture medium (Figure 7C) upon VPS35 siRNA treatment. Further Wnt3a secretion was impaired upon the knockdown of VPS35 [14,15,18]. In addition, internalized Wls is likely to be sorted into lysosomes for degradation in the absence of retromer [18]. In support of this view, our data showed that the level of GPR177 was reduced when VPS35 was suppressed by VPS35 siRNA treatment (Figure 7D). In Wnt target cells, secreted Wnt protein interacts with the receptors Frizzled and LRP5/6 to activate the β-catenin pathway [7]. A previous study showed an indirect interaction between the LRP6 and VPS35 in HEK-293 cells [29], however, a possible interaction between the LRP6 and VPS35 in MSCs has not been reported.

V-ATPases regulate pH in acidic subcellular compartments including the Golgi complex and lysosomes. Wls-dependent secretion of Wnt3a requires vacuolar acidification [19]. In the presence of acidification inhibitors, the Wnt3a-Wls complex is able to reach the cell surface but the release of Wnt3a from Wls is hindered [19]. Treatment of cells with siRNA targeting two subunits of V-ATPase (ATP6V1 and ATP6V0) inhibited Wnt signaling [12]. Although we found that HBO treatment increased Wnt3a production, the increased Wnt3a protein was retained in the producing cells (Figure 8B) and was unable to move into the culture medium (Figure 8C) during ATP6V0 siRNA treatment.

Long-term and repeated HBO treatments may increase oxidative stress; however, tolerance to HBO treatment can be extended by intermittent exposure [30]. Because exposure to HBO in clinical protocols is rather brief (typically <2 h/d), studies show that antioxidant defenses are adequate so that stresses related to increases in ROS are reversible [30]. In addition, HBO treatment was shown to suppress the apoptosis in degenerated disc cells [31] and osteoarthritic chondrocytes [32], suggesting a beneficial effect of HBO. Taken together, our results suggested that HBO increases osteogenic differentiation of MSCs via regulation of Wnt processing, secretion, and signaling. Further understanding of the regulatory factors and molecular mechanism involved, HBO may serve as a therapeutic approach to increase bone healing in clinical setting.

## Conclusions

HBO treatment increased osteogenic differentiation of MSCs via regulating Wnt processing, secretion, and signaling.

## Competing interests

The authors declare that they have no competing interests.

## Authors’ contributions

Study design: LSS, USWN, CJK. Data analysis and interpretation: LSS, YCY, LMS. Provision of study material or patients: NCC, YLJ, CWJ. Drafting manuscript: LSS, USWN, CJK. All authors approving final version of manuscript.

## Authors’ information

Song-Shu Lin is submitting author.

## Pre-publication history

The pre-publication history for this paper can be accessed here:

http://www.biomedcentral.com/1471-2474/15/56/prepub
